# Transformation Characteristics of Hydrogen-Donor Solvent Tetralin in the Process of Direct Coal Liquefaction

**DOI:** 10.3389/fchem.2019.00737

**Published:** 2019-11-01

**Authors:** Hai-zhou Chang, Jun-qi Li, Shuai Du, Kai-yuan Shen, Qun Yang, Han Yi, Ji-wei Zhang

**Affiliations:** ^1^College of Science, University of Shanghai for Science and Technology, Shanghai, China; ^2^SGS-CSTC Standards Technical Services Co., Ltd, Shenzhen, China; ^3^School of Mechanical Engineering, University of Shanghai for Science and Technology, Shanghai, China

**Keywords:** coal liquefaction, tetralin, oil analysis, Gas analysis, transformation (of products)

## Abstract

The aim of this study is to investigate the transformation of hydrogen-donor solvent tetralin in the direct liquefaction process of coal. Pure tetralin liquid as well as mixture of tetralin and Wucaiwan coal (WCW) were separately reacted under a liquefaction condition, and constituents of liquid product were analyzed by GC-MS. The results show that after the tetralin liquid reacts with high-pressure hydrogen, 90% of the reaction product is in liquid state, the gaseous products mainly include alkane gas and CO_x_ gas. When the reaction temperatures were set at 380 and 420°C, respectively, the corresponding transformation rates of tetralin can be 34.72 and 52.74%. At 380°C, the tetralin mainly plays a role of passing active hydrogen, while at 420°C, it mainly occurs dehydrogenation transformation to provide active hydrogen, as well as generate naphthalene, methyl indan, and substituted benzene, etc. Taking tetralin as the hydrogen-donor solvent, the WCW was performed liquefaction reaction, and the obtained results show that the transformation rates of tetralin are 69.76 and 83.86% at liquefaction temperatures of 380 and 420°C, respectively. Tetralin mainly occur to dehydrogenation transformation to generate naphthalene, followed by methyl indan, where contents order of main constituents of the liquefaction products were: naphthalene> tetralin > methyl indan.

## Introduction

Direct liquefaction of coal is an effective measure for clean utilization of coal, which converts solid coal (H/C ratio ≈ 0.8) to liquid fuels (H/C ratio ≈ 2) by adding hydrogen (Ren et al., 2010; Vasireddy et al., 2011). It is vital to provide enough hydrogen in a timely manner to stabilize free radicals from coal pyrolysis for getting more liquid fuels and inhibiting coke formation in the direct liquefaction process (Shui et al., 2010). It has been found solvent as a media plays a very important role in liquefaction process of coal (Mochida et al., 1990; Xue et al., 1999; Yan et al., 2001; Zhang et al., 2009), such as dissolving, providing, and passing active hydrogen as well as stabilizing free radicals, etc. Studies have revealed that tetralin is a kind of excellent hydrogen-donor solvent (Neavel, 1976; Ishihara et al., 2002; Shu et al., 2003; Li and Ling, 2007; Zhang, 2011; Hou et al., 2018), where it can well dissolve and disperse coal particles, has strong ability to donate hydrogen as well as quickly stabilize free radicals, etc. Hydrogen transferred from tetralin affects oil yield and coal conversion significantly in the preheating stage of coal liquefaction (Hao et al., 2017). Approximately same total hydrogen consumption is observed under N_2_ and H_2_ with tetralin, and the hydrogen consumption from tetralin is higher than that from H_2_ (Hao et al., 2018). It should be noted that tetralin is generally selected as hydrogen-donor solvent in direct liquefaction of coal conducted in laboratories. However, part of the tetralin tends to transform under the liquefaction conditions (Zhang et al., 2009). Since the specific transformation of tetralin is associated with the calculation of liquefaction product yield and understanding of liquefaction mechanism, knowing the transformation characteristics of tetralin during direct liquefaction process is a basic question that needs to be addressed. Yang et al. (1985a,b) studied the pyrolysis process of tetralin and found that the tetralin mainly leads to dehydrogenation reaction to generate naphthalene and isomerization reaction to generate methyl indan. Furthermore, they proposed the mechanism of radical reaction. Yang et al. (2011) summarized the reaction mechanism and reaction dynamics in tetralin hydrogenation pyrolysis. Ishihara et al. (2002) applied isotope labeling method to study the hydrogen transfer behaviors between coal and tetralin in direct liquefaction process, and they obtained the hydrogen donating pathways: I. Directly transferring active hydrogen; II. Transforming to naphthalene by dehydrogenation. Studies have reported the mechanism of tetralin pyrolysis as well as hydrogen donating behavior of tetralin in direct liquefaction process using isotope label method, but the transformation of tetralin itself in direct liquefaction process is still unknown. In this study, we obtained liquid products by separately performing reaction using pure tetralin as well as mixture of coal and tetralin under direct liquefaction condition. Then we utilized GC-MS to quantitatively analyze constituents of the liquid and changes of their contents, utilized GC to obtain the composition distribution in gas phase, hereby to study the transformation features of tetralin, so as to enrich the understanding of the mechanism of direct coal liquefaction.

## Materials and Methods

### Materials

A Chinese WCW coal was used. Its ultimate and proximate analyses are listed in Table 1. The coal sample was ground to pass 200 mesh sieve and dried under a vacuum at 105°C for 4 h. Tetralin (AR) and Hexane (AR) provided by Sinopharm Chemical Reagent Co., Ltd were used in the experiments.

**Table 1:** Proximate analysis and ultimate analysis of WCW coal.

**Sample**	**Proximate**		**Analysis**	**Ultimate**		**Analysis**		**(wt.%, daf)**
	**M_**ad**_**	**A_**ad**_**	**V_**daf**_**	**C**	**H**	**N**	**S**	**O**
WCW	12.28	6.11	31.13	79.75	3.55	0.63	0.59	15.48

### Direct Liquefaction Experiments

#### Coal Direct Liquefaction Procedure

Two grams of WCW coal (200 mesh) and 4 g tetralin were weighted and transferred to the autoclave, added with catalyst (Fe_2_O_3_) (iron amount: 3 wt % of coal was transformed to Fe_2_O_3_) and co-catalyst S (0.4 times of Fe mass). The initial pressure of hydrogen was 5 MPa, and the temperature was set to be 380 and 420°C, respectively. The reaction was persisted for 60 min after obtaining the pre-set temperature, and then the experiment was terminated. Gas was collected after the autoclave cooled to room temperature. After the autoclave was opened, the oil and residue were fully transferred to paper-cylinder filtration and placed into an extraction tube, which was placed into a flask containing 200 g hexane, and refluxed in 107°C silicon oil bath pan for 48 h, hereby to obtain orange oil. Constituents of gas were analyzed using gas chromatograph and those of oil were analyzed using gas chromatograph-mass spectrometer.

#### Tetralin Direct Liquefaction Procedure

Four grams of tetralin were weighted and transferred to the autoclave, added with catalyst (Fe_2_O_3_) [iron amount: 3 wt % of coal (2 g) was transformed to Fe_2_O_3_] and co-catalyst S (0.4 times of Fe mass). The initial pressure of hydrogen was 5 MPa, and the temperature was set to be 380 and 420°C, respectively. The reaction was persisted for 60 min after obtaining the pre-set temperature, and then the experiment was terminated. Gas was collected after the autoclave cooled to room temperature. After the autoclave was opened, the oil and residue were fully transferred to paper-cylinder filtration and placed into an extraction tube, which was placed into a flask containing 200 g hexane, and refluxed in a 107°C silicon oil bath pan for 48 h, hereby to obtain orange oil. Constituents of gas were analyzed using gas chromatograph and those of oil were analyzed using gas chromatograph-mass spectrometer.

### GC/MS Analysis

GC-MS Thermo Trace GC ISQ was used to analyze liquid phases in the experiments. Its key parameters include TR-5MS capillary column (length 30 m, inner diameter 0.25 mm, film thickness 0.25 μm); carrier gas: helium, flow rate 1.5 mL/min; split flow 20:1; temperature of injection port 280°C; EI source, ionization voltage 70 eV, temperature of ion source 275°C; mass scanning range 50–550 amu. The elevation of temperature was programed as follows: initial temperature of 50°C (1 min), and elevated to 90°C (5°C/min, 2 min) and elevated to 240°C (10°C/min, 1 min). The constituents of the obtained oil were analyzed, and the relative percentage of each constituent was calculated using area normalization method.

## Results and Discussion

### Composition of Products of Tetralin and High-Pressure Hydrogen

Liquid, gas, and residue yield were calculated by equations shown as below,

Liquid yield: Y_l_ = M_l_/(M_t_ + M_h_)

Residue yield: Y_r_ = (M_r_ - M_cat._)/(M_t_ + M_h_)

Gas yield: Y_g_ = 1 - Y_l_ - Y_r_

Y_l_, Y_r_, Y_g_ are the yield of liquid, residue and gas. M_t_, M_h_, M_cat._ are weight of tetralin, molecular hydrogen, and catalyst (Fe_2_O_3_). M_l_, M_r_ are weight of liquid and residue after the reaction.

Table 2 lists the contents of gas, liquid, and solid obtained by tetralin under high-pressure hydrogen reaction at 380 and 420°C. At the designated temperature, contents of each constituent were ordered by: liquid >gas>solid, of which the contents of liquid constituent were 92.14 and 85.76%, respectively, which indicated that the tetralin is mainly in a liquid state during the reaction process, while part of it is transformed into gas and solid. Comparing products obtained at two temperatures revealed that with increasing temperature, content of liquid constituents was decreased and contents of gas and solid constituents were increased. Based on the above results, it indicated that an elevated temperature is prone to aggravate the liquid tetralin to transform to gas and solid products. Thus, it can be inferred that in direct coal liquefaction experiment using tetralin as hydrogen-donor solvent, part of tetralin will transform to gas and solid. Since the coal liquefaction requires a large amount of tetralin, effect of tetralin transformation on products of coal liquefaction is worthy of concern.

**Table 2:** Contents of products obtained by tetralin and high-pressure hydrogen reaction.

**Temperature**	**Yield/%**		
	**Liquid**	**Gas**	**Residue**
380°C	92.14	4.93	2.93
420°C	85.76	8.39	5.85

### GC-MS Analysis of Liquid Obtained by Tetralin and High-Pressure Hydrogen Reaction

#### GC-MS Analysis of Pure Tetralin

In this paper, the transformation characteristics of tetralin were discussed on the basis of comparative analysis. In order to make the analysis results more reliable and accurate, the components of tetralin solvent were analyzed by GC-MS. Results (Table 3) show that 97.04% of the tetralin solvent exists in the form of tetralin, 2.62% in the form of non-tetralin, and 0.34% in the form of unknown components. The blank analysis of tetralin solvent is helpful to further study the transformation mechanism of tetralin in the liquefaction process of tetralin and coal liquefaction process using tetralin as hydrogen-donor solvent.

**Table 3:** Composition of the raw material tetralin.

**NO**.	**Compound**	**Molecular formula**	**Area%**
1	Tetralin	C_10_H_12_	97.04
2	Naphthalene	C_10_H_8_	1.09
3	Methyl indan	C_10_H_12_	0.46
4	Butadiene styrene	C_10_H_14_	0.15
5	Ethylbenzene	C_8_H_10_	0.27
6	m-xylene	C_8_H_10_	0.22
7	Ethyltoluene	C_9_H_12_	0.28
8	n-decane	C_10_H_22_	0.15

#### GC-MS Analysis of Oil Products Obtained by Tetralin and High-Hydrogen Reaction

**Scheme 1:**
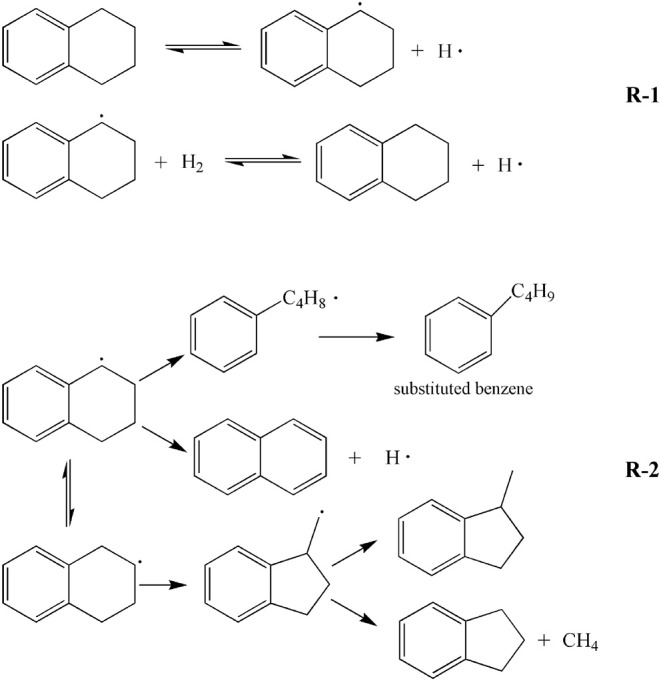
Possible mechanism of hydrogen transfer or dehydrogenation of tetralin in direct liquefaction process.

Table 4 shows GC-MS analysis results of liquid obtained by tetralin and high-pressure hydrogen reaction, which demonstrated that at 380°C, contents of the main constituents of the liquid were ordered by: tetralin>naphthalene>methyl indan, of which the percentage of tetralin was 65.28%, which was significantly lower than the GC-MS analysis results of 97.04% of pure tetralin liquid. The reason is that the tetralin transforms to generate naphthalene (13.71%), methyl indan (12.48%), and substituted benzene (6.77%), as well as a small amount of straight-chain alkanes and methylnaphthalene, etc. At 420°C, contents of the main constituents of the liquid were ordered by: tetralin> methyl indan> naphthalene, of which the content of tetralin was only 47.26%, while the contents of other constituents were increased. In details, the content of methyl indan was significantly increased to 20.80%, the content of naphthalene was increased to 15.05%. In addition, the content of substituted benzene was increased from 6.77 to 11.62%. These results indicated that an elevated temperature tends to enhance the transformation of tetralin, and tetralin is mainly transformed into methyl indan, followed by naphthalene at 420°C. In summary, analysis of constituents of the liquid showed that in the reaction process of tetralin and high-pressure hydrogen, tetralin could play a role of transferring hydrogen as illustrated in formula R-1 shown in Scheme 1, which was denoted as THN_1_, meanwhile it could also provide hydrogen through its own dehydrogenation transformation as illustrated in formula R-2 shown in Scheme 1, which was denoted as THN_2_. From Table 2, the transformation rates of tetralin were 34.72 and 52.74%, and the THN_1_/THN_2_ was 1.88 and 0.90 at 380 and 420°C, respectively. These findings revealed that at a lower reaction temperature (such as 380°C), the hydrogen-donor solvent tetralin mainly plays a role of transferring active hydrogen. However, at a higher reaction temperature (such as 420°C), tetralin mainly provides active hydrogen through its own dehydrogenation transformation.

**Table 4:** Relative content of hydrocarbons in liquid products.

**No**.	**Compound**	**Molecular formula**	**Area%**
**380°C**			
1	Tetralin	C_10_H_12_	65.28
2	Naphthalene	C_10_H_8_	13.71
3	Methyl indan	C_10_H_12_	12.48
4	Butylbenzene	C_10_H_14_	5.30
5	Ethylbenzene	C_8_H_10_	1.18
6	n-heneicosane	C_21_H_44_	0.48
7	Indane	C_9_H_10_	0.47
8	n-eicosane	C_20_H_42_	0.45
9	Hexadecane	C_16_H_34_	0.18
10	Methylnaphthalene	C_11_H_10_	0.17
11	Diethylbenzene	C_10_H_14_	0.16
12	m-xylene	C_8_H_10_	0.07
13	Ethyltoluene	C_9_H_12_	0.06
**420°C**			
1	Tetralin	C_10_H_12_	47.26
2	Naphthalene	C_10_H_8_	15.61
3	Methyl indan	C_10_H_12_	20.80
4	Butylbenzene	C_10_H_14_	5.68
5	Ethylbenzene	C_8_H_10_	4.95
6	Indane	C_9_H_10_	3.59
7	Methylnaphthalene	C_11_H_10_	0.57
8	n-eicosane	C_20_H_42_	0.42
9	Diethylbenzene	C_10_H_14_	0.30
10	Isopropyl benzene	C_9_H_12_	0.29
11	m-xylene	C_8_H_10_	0.28
12	Ethyltoluene	C_9_H_12_	0.12
13	Tridecane	C_13_H_28_	0.09

#### GC-MS Analysis of Oil Products Obtained by Liquefaction of WCW

Table 5 shows the GC-MS analysis of the oil products obtained by direct liquefaction of WCW, while taking tetralin as exxon donor solvent. As can be seen from the table, at a liquefaction temperature of 380°C, contents of the main constituents of oil were ordered by: naphthalene>tetralin>methyl indan, of which the content of naphthalene was highest, accounting for 58.67% of the total amount, while the content of tetralin was 26.42% (transformation rate of tetralin was 69.76%). At a liquefaction temperature of 420°C, contents of the main compositions of oil were still ordered by: naphthalene>tetralin>methyl indan, of which the content of naphthalene was as high as 65.96%, while the content of tetralin was only 14.86% (transformation rate of tetralin was 83.86%), which can be ascribed to the tetralin occurs dehydrogenation transformation to generate naphthalene, methyl indan, and other products.

**Table 5:** Relative content of hydrocarbons in oil.

**No**.	**Compound**	**Molecular formula**	**Area%**
**380°C**			
1	Naphthalene	C_10_H_8_	58.67
2	Tetralin	C_10_H_12_	26.42
3	Methyl indan	C_10_H_12_	5.49
4	Alkanes	C_8_-C_20_	3.56
5	Butylbenzene	C_10_H_14_	1.68
6	Methylnaphthalene	C_11_H_10_	1.15
7	Indane	C_9_H_10_	0.94
8	Ethylbenzene	C_8_H_10_	0.65
9	Pyrene	C_16_H_10_	0.36
10	Anthracene	C_14_H_10_	0.30
11	Fluorene	C_13_H_10_	0.20
12	Phenylpropane	C_9_H_12_	0.20
13	Acenaphthene	C_12_H_8_	0.18
14	2-methylbiphenyl	C_13_H_12_	0.18
**420°C**			
1	Naphthalene	C_10_H_8_	65.96
2	Tetralin	C_10_H_12_	14.86
3	Methyl indan	C_10_H_12_	7.12
4	Alkanes	C_13_-C_20_	4.04
5	Indane	C_9_H_10_	2.16
6	Methylnapht halene	C_11_H_10_	1.77
7	Ethylbenzene	C_8_H_10_	1.40
8	Butylbenzene	C_10_H_14_	1.00
9	Pyrene	C_16_H_10_	0.36
10	Anthracene	C_14_H_10_	0.24
11	Fluorene	C_13_H_10_	0.21
12	Phenylpropa ne	C_9_H_12_	0.30
13	Acenaphthene	C_12_H_8_	0.14
14	2-methylbiphenyl	C_13_H_12_	0.43

As shown in Table 6, the oil yield of WCW at liquefaction temperature of 380 and 420°C were 29.30 and 17%, respectively. In liquefaction reaction, coal macromolecules can decompose to generate part of the naphthalene in the oil. Mass conservation proves that the naphthalene in the oil mainly derives from transformation of tetralin. Assuming the percentages of tetralin, naphthalene and methyl indan in oil product were denoted as x, y, and z, respectively, while the total mass of oil product obtained by liquefaction was m_o_+m_T_, we might obtain the masses of tetralin, naphthalene and methyl indan as (m_o_+m_T_) ·x, (m_o_+m_T_) ·y and (m_o_+m_T_)·z, respectively. Table 7 shows the balancing results of tetralin in oil product obtained by liquefaction, from the Δ value, reduction of tetralin is about the summation of masses of naphthalene and 2,3-indanyl, hereby proves that the naphthalene in oil product is mainly transformed from tetralin.

**Table 6:** Oil yield, transformation rate, and residue rate in liquefaction of WCW.

**Temperature**	**η_oil_/%**	**η_conv_/%**	**η_char_/%**
380°C	29.30	48.42	51.58
420°C	17.00	48.15	51.84

**Table 7:** Balancing results of mass of tetralin in oil product obtained by liquefaction (Assuming: under liquefaction conditions, the tetralin is completely in the liquid state and its mass is denoted as m_T_, while the mass of oil product from coal transformation is denoted as m_o_).

**Temperature**	**m_T_ - (m_o_+m_T_) ·x**	**(m_o_+m_T_) · y+(m_o_+m_T_)·z**	**Δ**
380°C	2.82	3.02	−0.20
420°C	3.32	3.23	0.090

In addition, the main constituents of oil product obtained by liquefaction of WCW include tetralin, naphthalene, and methyl indan, and their contents were ordered by: naphthalene> tetralin> methyl indan. Therefore, it could be inferred that the tetralin was mainly transformed into naphthalene and methyl indan in liquefaction reaction of WCW, which was consistent with the constituents of oil obtained by reaction of tetralin and high-pressure hydrogen. In this study, liquefaction temperatures of 380 and 420°C were selected. Constituents of oil at these two temperatures were compared, and the results showed that the tetralin in the oil was significantly decreased with elevating temperature, which was reduced from 26.42 to 14.86%, and the content of naphthalene was increased from 58.67 to 65.96%, while the content of methyl indan was slightly increased from 6.43 to 9.28%. In addition, by comparison of the data in Tables 3, 4, the results revealed that the main constituents of oil presented a change of “two decrease, one increase,” i.e., contents of tetralin and methyl indan were decreased, while content of naphthalene was significantly increased. Therefore, it can be inferred that during the liquefaction of WCW, the hydrogen-donating paths for the tetralin are: (a) Mainly, it is a process for tetralin occurs dehydrogenation reaction to produce naphthalene; (b) C-C bond of tetralin molecules tends to break and reconstruct to generate isomer methyl indan, part of which occurs demethylation reaction to produce indan and methane gas; (c) C-C bond at α site of tetralin molecules tends to cleavage into benzene butyl free radicals, and then transforms to substitute benzene and paraffin gas; (d) Tetralin molecules directly transfer active hydrogens as illustrated in formula (1). Overall, during the liquefaction process of WCW, the hydrogen-donating path of hydrogen-donator solvent was similar to the reaction process of tetralin and high-pressure hydrogen. It is worth noting that the content of naphthalene was significantly higher than the contents of methyl indan and tetralin in oil obtained by liquefaction of WCW. The reason is that the path (a) is the main transformation process, and the naphthalene has a weaker hydrogen-donating ability and has a better thermal stability.

Compared with constituents of liquid obtained by reaction of tetralin and high-pressure hydrogen, the oil products obtained by coal liquefaction contain benzo pyrene, anthracene, fluorene, acenaphthene, and other polycyclic aromatic hydrocarbon structures. This part of aromatic constituents is mainly produced by coal macromolecule, i.e., at reaction temperature, weak bonds in coal macromolecule structure will break to produce aromatic free radicals, which bond to active hydrogen to produce aromatic compound.

### Constituents of Gas Obtained by Tetralin and High-Pressure Hydrogen Reaction

GC-9160 gas chromatograph was used to analyze the gas obtained by reaction of tetralin and high-pressure hydrogen, and the results (Table 8) showed low contents of alkane gas and CO_x_ gas. For CO_x_ gas, the distribution was characterized by CO_2_ >CO at two reaction temperatures. For alkane gas, contents of gas were ordered by CH_4_ >C_2_H_6_ >C_3_H_8_ >C_4_H_10_ and C_2_H_6_ >CH_4_ >C_3_H_8_ >C_4_H_10_ at reaction temperature of 380 and 420°C, respectively. From the point of view of conservation of carbon atoms, both alkane and CO_x_ gases are transformed from tetralin. In summary, among the gas obtained by reaction of tetralin and high-pressure hydrogen, the content of alkane gas was greater than that of CO_x_ gas, where contents of the former were 2.4 and 4.5 times of those of the latter at 380 and 420°C, respectively, which in a whole presented distribution characteristics of rich alkanes, methane, and carbon dioxide.

**Table 8:** Constituents of gas obtained by tetralin and high-pressure hydrogen reaction.

**Tetralin**	**CH_4_**	**C_2_H_6_**	**C_3_H_8_**	**C_4_H_10_**	**CO**	**CO_2_**
380°C	0.49	0.27	0.060	0.010	0.10	0.24
420°C	0.42	0.50	0.070	0.010	0.090	0.13

## Conclusion

In conclusion, when the pure tetralin liquid reacts at liquefaction conditions, the tetralin mainly exists in a liquid state, while part of it may transform into gas and solid. In addition, the content of liquid constituent is decreased while the contents of gas and solid constituents are increased with elevating temperature.

In the reaction process of tetralin and high-pressure hydrogen, part of tetralin plays a role of transferring hydrogen, and the remaining part tends to provide hydrogen through its own dehydrogenation transformation. At the lower temperature (such as 380°C), tetralin mainly plays a role of transferring active hydrogen with a transformation rate of 34.72%. Meanwhile, at a higher temperature (such as 420°C), tetralin mainly provides active hydrogen through dehydrogenation transformation and has a transformation rate of 52.74%, while it can transform into naphthalene, methyl indan, and substituted benzene, etc. In addition, gas phase produced from the reaction generally presents distribution of high concentration of alkanes, methane, and carbon dioxide, and both the alkane and CO_x_ gases are transformed from tetralin.

As the hydrogen-donor solvent, the transformation rates of tetralin are 69.76 and 83.86% in liquefaction of WCW at temperatures of 380 and 420°C, respectively. The contents of main constituents of oil are ordered by: naphthalene>tetralin> methyl indan. In addition, compared with the oil product obtained by liquefaction of pure tetralin, content of tetralin in oil product obtained by liquefaction of coal is significantly reduced. Meanwhile, the content of methyl indan is decreased. However, the content of naphthalene is significantly increased.

## Data Availability Statement

All datasets generated for this study are included in the article/supplementary material.

## Author Contributions

All authors listed have made a substantial, direct and intellectual contribution to the work, and approved it for publication.

### Conflict of Interest

SD has been employed by company SGS-CSTC Standards Technical Services Co., Ltd. The remaining authors declare that the research was conducted in the absence of any commercial or financial relationships that could be construed as a potential conflict of interest.
